# Serum snoRNAs as biomarkers for joint ageing and post traumatic osteoarthritis

**DOI:** 10.1038/srep43558

**Published:** 2017-03-02

**Authors:** Mandy M. F. Steinbusch, Yongxiang Fang, Peter I. Milner, Peter D. Clegg, David A. Young, Tim J. M. Welting, Mandy J. Peffers

**Affiliations:** 1 Department of Orthopedic Surgery, Caphri School for Public Health and Primary Care, Maastricht University Medical Center, Maastricht, the Netherlands; 2 Centre for Genomic Research, Institute of Integrative Biology, Biosciences Building, Crown Street, University of Liverpool, Liverpool L69 7ZB, UK; 3 Institute of Ageing and Chronic Disease, University of Liverpool, Apex Building, 6 West Derby Street, Liverpool, L7 9TX, UK; 4 Musculoskeletal Research Group, Newcastle University, Newcastle upon Tyne NE2 4HH, UK

## Abstract

The development of effective treatments for the age-related disease osteoarthritis and the ability to predict disease progression has been hampered by the lack of biomarkers able to demonstrate the course of the disease. Profiling the expression patterns of small nucleolar RNAs (snoRNAs) in joint ageing and OA may provide diagnostic biomarkers and therapeutic targets. This study determined expression patterns of snoRNAs in joint ageing and OA and examined them as potential biomarkers. Using SnoRNASeq and real-time quantitative PCR (qRT-PCR) we demonstrate snoRNA expression levels in murine ageing and OA joints and serum for the first time. SnoRNASeq identified differential expression (DE) of 6 snoRNAs in young versus old joints and 5 snoRNAs in old sham versus old experimental osteoarthritic joints. In serum we found differential presence of 27 snoRNAs in young versus old serum and 18 snoRNAs in old sham versus old experimental osteoarthritic serum. Confirmatory qRT-PCR analysis demonstrated good correlation with SnoRNASeq findings. Profiling the expression patterns of snoRNAs is the initial step in determining their functional significance in ageing and osteoarthritis, and provides potential diagnostic biomarkers and therapeutic targets. Our results establish snoRNAs as novel markers of musculoskeletal ageing and osteoarthritis.

Osteoarthritis (OA) is an age-related musculoskeletal disease and a common cause of chronic disability worldwide^1^. In addition it is a significant contributor to both individual and socioeconomic burden and the number of disability adapted life years globally^2^. If the deterioration in musculoskeletal health and development of OA can be identified and treated early serious life impairment may be abrogated. Ageing is the time-dependent reduction of functional capacity and stress resistance, associated with an increased risk of morbidity and mortality. The joint and its articular cartilage is particularly affected by ageing^3^. There is evidence that the rate of ageing, that is the ‘biological age’, differs significantly between individuals’ actual age in years (i.e. the ‘chronological age’). Defining markers of joint ageing may enable a prediction of the risk of onset of OA, enabling early intervention. OA is characterised by a non-symptomatic, pre-radiographical phase that if identified would allow earlier diagnosis. However radiographic changes are only evident later in disease progression. Magnetic resonance imaging techniques have been developed for early-stage evaluation of cartilage damage in OA but are expensive and contraindicated in some individuals.

The development of effective treatments for OA and the ability to predict disease progression has been hampered by the lack of substantive biomarkers, able to demonstrate pathological disturbances preceding identifiable tissue alterations. Others have attempted to identify products of tissue turnover in serum and synovial fluid (reviewed^4^). This has been challenging due to patient and disease heterogeneity and dilution effects either by tissue fluids or with similar products from other joints or diseases. In addition, the variability of antibody assays has been problematic.

SnoRNAs are a class of evolutionary conserved non-coding small guide RNAs of which the majority direct the chemical modification of other RNA substrates, including ribosomal RNAs and spliceosomal RNAs. In addition, some snoRNAs are involved in the regulation of alternative splicing and post-transcriptional modification of mRNA, whilst others exhibit miR-like activity^5^. Aberrant expression of snoRNAs has been associated with disease development^5^ such as lung tumorigenesis^6^.

Emerging evidence shows that there is an increased level of circulating RNAs in the serum of cancer patients^7^. Circulating microRNAs (miRs) have been extensively described as biomarkers for diseases like pancreatic/breast cancer^8^,^9^, Alzheimer’s disease^10^ and inflammatory diseases like asthma, inflammatory bowel disease and rheumatoid arthiritis^11^, but with the recent discovery of stable^12^ snoRNAs in serum, interest in their potential as circulating biomarkers of cancers (reviewed^5^) has been stimulated. We have previously identified dysregulation of a defined set of snoRNAs in cartilage^13^ and tendon^14^ ageing and OA^15^ and in man, snoRNA SNORD38 and SNORD48 were identified as potential non-age-dependant serum biomarkers for OA progression following cruciate ligament injury^12^.

Expression profiling of snoRNAs in ageing and OA may help in determining their functional significance in the development and progression of disease and provide much needed diagnostic biomarkers for ageing and OA development. This study compared serum and joint snoRNA expression in ageing and OA from knee joint tissues from young and old adult mice and old mice using a traumatic *in vivo* model of OA. Because OA involves the whole joint as an organ; we undertook our analysis on whole mouse joints, which included cartilage, meniscus, subchondral bone, and joint capsule with synovium.

## Materials and Methods

All reagents were from Thermo-Fisher-Scientific, unless stated.

### Animals

C57BL6/J male mice were used for the study. For SnoRNASeq old mice were 18 months old (n = 6), young 8 months old (n = 6)^16^ and mice used for destabilisation of the medial meniscus (DMM) 24 months old (sham n = 3; DMM n = 6). Mice were group housed in individually ventilated cages at a 12 hour light/dark cycle, with *ad libitum* access to food and water. Experimental animal protocols were performed in accordance with the guidelines of the Animals (Scientific Procedures) Act 1986 following ethical review. Animal usage and protocols for this study was approved by the University of Liverpool Animal Welfare Committee.

### Surgical induction of OA by DMM in mice

DMM surgery was perform as previously reported^17^. Briefly, under anaesthesia a 3 mm skin incision was made over the medial aspect of the patellar ligament through the joint capsule into the femorotibial joint of the left knee. The medial meniscotibial ligament was transected to destabilise the cranial pole of the medial meniscus from the anterior tibial plateau. In sham operated mice the medial meniscotibial ligament was visualised but not transected. Mice were sacrificed 8 weeks post-surgery.

### Joint and serum collection for SnoRNASeq

Following euthanasia, knee joints were collected from young, old, DMM (n = 6 each group) and sham n = 3 for SnoRNASeq. Joints were harvested free of soft tissues at 7 mm from the joint into RNALater. Serum was collected using cardiac puncture. One old serum sample was not processed further due to extensive haemolysis.

### OARSI scoring of histological sections of mouse knee joints

For histology, as the total knee joint was used in the study for RNA extraction, joints were collected (into 4% paraformaldehyde) from additional equivalent aged and treated young (n = 8), old (n = 4); sham (n = 5); and DMM (n = 6) mice in order to evaluate the extent of OA. The procedure, surgeon and duration of the studies were identical. Knees were decalcified in 0.5 M ethylenediaminetetraacetic acid (pH 7.4) for 4 weeks at 4 °C and coronally embedded in paraffin. Sectioning, Safranin-O Fast-Green staining and histological scoring (defined as the severity and extent of OA) was undertaken on a scale from 1 to 6 by two blinded independent observers using the OARSI histopathology initiative^18^. All four quadrants of the section (medial tibial plateau, lateral tibial plateau, medial femoral condyle, lateral femoral condyle) were scored individually and added for each histological section. For statistical analyses mean summed score values of joints of 3–5 section per knee joint at 4 depths throughout the joint was determined (thus a maximum score of 24 was possible). Inter-observer variability was calculated using Cohen’s Kappa statistics using an online software tool: (http://www.statstodo.com/CohenKappa).

### RNA isolation, RNA-Seq analysis, cDNA library preparation and sequencing

Total RNA was isolated from equal weights of joints and 500 μl serum using miRNeasy or RNeasy Serum kits respectively with DNase treatment (all Qiagen, Crawley, UK) to remove residual gDNA. Total RNA integrity (RIN) was confirmed using the Agilent 2100 Bioanalyzer (Agilent Technologies, Santa Clara, USA). Ribosomal RNA was depleted using the Ribo-Zero™ rRNA Removal Kit (Epicentre, Madison, USA). From 41 samples 100 ng of rRNA-depleted RNA was submitted for library preparation using NEB small RNA library kit (New England Biolabs (NEB), Ipswich, USA). To reduce workflow bias we used tobacco acid pyrophosphatase (Epicentre, Madison, USA) to remove potential 5′ caps found on some snoRNAs. Samples were amplified for 15 cycles, mixed into 3 pools, and size selected. The size-selected material was purified with Ampure beads (Agencourt, Beckman-Coulter, High-Wycombe, UK). SnoRNA sequencing was undertaken on the Illumina HiSeq 2000 platform (Illumina, San Diego, USA) using 100 base paired-end reads.

### SnoRNASeq data analysis

Sequence data measured from 5 lanes of an Illumina HiSeq2000 were processed through a number of steps to obtain snoRNA expression values. The processes include basecalling and de-multiplexing of indexed reads using CASAVA version 1.8.2^19^; adapter and quality trimming using Cutadapt version 1.2.1^20^ and Sickle version 1.200 to obtain fastq files of trimmed reads; aligning reads to Ensembl GRCm38.77 mouse genome reference sequences which contains 1,555 annotated snoRNA features using Bowtie2^21^ version 2.0.10 with option “–very-sensitive-local”; counting aligned reads against snoRNA features using THSeq-count. The count values were used as snoRNA expression measurements for the DE analysis.

DE analysis was performed in R environment using package edgeR^22^. The processes and technical details of the analysis include: assessing data variation and detecting outlier samples through comparing variations of within and between sample groups using principle component analysis (PCA; 3-D PCA plots were generated using R function in package plot3D) and correlation analysis; handling library size variation respectively for joint samples and serum samples through data normalisation; formulating data variation using negative binomial distributions; modelling data using a generalised linear model; computing log_2_ Fold Change (logFC) values for required contrasts based on model fitting results through contrast fitting approach, assigning P-values to logFC values by LR^23^ testing; dealing with the effects of multiple tests using FDR approach to obtain FDR adjusted P-values; and defining significantly DE snoRNAs as those with FDR-adjusted p-value < 5%. Sequence data have been submitted to National Centre for Biotechnology Information Gene Expression Omnibus (NCBI GEO); E-MTAB-4878.

### RNA isolation, poly(A) cDNA synthesis and snoRNA qRT-PCR

qRT-PCR of snoRNAs was performed^24^. Total RNA was isolated using the mirVana kit. Isolated RNA samples were polyadenylated at 37 °C for 60 minutes in a 50 μL reaction volume containing 1 μg RNA and 1.5 U poly(A) polymerase (NEB, Ipswich, USA). 500 μL lysis binding buffer was added. Then, an equal volume of acid-phenol:chloroform was added, vortexed and samples were centrifuged for 10 minutes and the aqueous phase removed. The poly(A)-tailed total RNA was extracted using the filter cartridge provided by the mirVana kit. To generate poly(A) cDNA, 500 ng poly(A)-tailed RNA and 250 ng RTQ primer (Eurogentec, Seraing, Belgium) (Table 1) were mixed in a 26 μL reaction volume, incubated at 65 °C for 10 minutes and annealed at 4 °C for 20 minutes. Reverse transcription was performed with 200 U M-MLV reverse transcriptase, 20 U RNAsin (both Promega, Southampton, UK), 2 μL dNTP mix (10 mM each; Eurogentec, Seraing, Belgium) and 8 μL 5x M-MLV buffer (Promega, Sothampton, USA) in a total reaction volume of 40 μL at 50 °C for 60 minutes. The reverse transcriptase was inactivated at 70 °C for 15 minutes. Finally, 1.5 U of RNAse H (NEB, Ipswich, USA) was added to remove small RNAs. A snoRNA-specific forward primer and a universal reverse primer (RTQ-UNIr, matched to the *T*m of each individual snoRNA) were used for the amplification of each snoRNA target (all Eurogentec, Seraing, Belgium) (Table 1). For each cDNA sample a mix was prepared with Mesagreen qPCR Mastermix Plus for SYBR Green (Eurogentec, Seraing, Belgium) and 300 nM forward and reverse oligonucleotides. An ABI-7300 Detection System was used for amplification using the following protocol: denaturation at 95 °C for 5 minutes, followed by 50 cycles of DNA amplification (15 seconds 95 °C and 45 seconds annealing at 62–68 °C). The annealing temperature was optimized for each snoRNAs target. Serially diluted standard curves were utilized to quantify snoRNA expression and data was normalized to a validated housekeeping snoRNA (joint: U2, young-old serum: SNORD85, old sham-old DMM serum and equine serum: U6).

### Validation of SNORD116 as a marker of OA in equine serum

We determined the reproducibility of the expression of SNORD116 in OA serum of another species. There are well-published studies on the application of metacarpophalangeal (MCP) joint OA changes in the horse, a joint with similarities to the human knee joint^25^. We studied equine serum from normal and MCP OA horses collected from eight normal (mean age ± standard deviation 5.3 ± 2.1 years) and four OA (7.5 ± 1.0 years) castrated male thoroughbred horses at post-mortem. Samples were collected under the regulations of the Hong Kong Jockey Club with owner consent and stored at −80 °C. OA diagnosis was based on histological (modified Mankin)^26^ and synovitis scoring^27^ of MCP joint tissues.

### Statistical analysis

For statistical evaluation of histological scoring non-parametric Mann-Whitney-U test was used. Inter-observer agreement of histological scoring systems was calculated using Cohen’s kappa coefficient (www.statstodo.com/CohenKappa_Pgm.phpl). qRT-PCR data was log-transformed prior to statistical evaluation with an independent samples t-test. Statistical evaluation was performed between young and old or old sham versus old DMM using GraphPad Prism 5 (San Diego); p-values are indicated.

## Results

### OARSI scoring of joints

**Figure 1:**
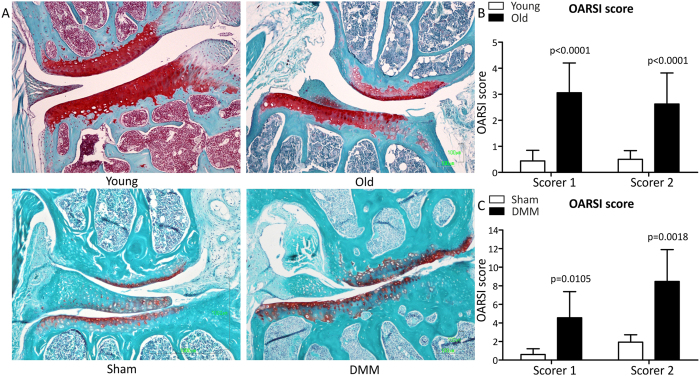
Histological changes of in the mouse knee showing the medial femoral condyle (above) and medial tibial plateau (below). (**A**) Safranin O with Fast-Green counterstain. Scale bar, 100 μm. Red indicates proteoglycan. (**B**/**C**) Assessment of osteoarthritis development was evaluated by OARSI scores; young (n = 8) vs. old (n = 4) (**B**); sham (n = 5) vs. DMM (n = 6) (**C**). Data represents the mean + 95% confidence interval (CI) for each scorer. For statistical evaluation an independent samples t-test was performed using GraphPad Prism 5 (San Diego); p-values are indicated.

OARSI scoring of joints (mean ± 95%CI) for young and old were 0.5 ± 0.3 and 2.8 ± 2.7 (p = 0.01), and old sham and old DMM were 1.25 ± 1.1 and 6.5 ± 0.7 (p < 0.001), respectively. Mice exhibited typical histological features of OA in the DMM knees. Cohen’s Kappa statistic was 0.4 indicating a fair agreement. Representative histological images and OARSI scoring are in Fig. 1.

### Preliminary analysis SnoRNASeq

To identify DE of snoRNAs in mouse joints and serum in response to age and OA 41 cDNA libraries representing old and young joints (old = HJO; young = HJY) and serum (old = HSO; young = HSY), and old sham and old DMM joints and serum (sham joint = DJC, DMM joint = DJM, sham serum = DSC and DMM serum = DSM) were constructed and subjected to Illumina deep sequencing. Summaries of raw, trimmed reads and sequencing alignment to mouse snoRNAs are in Supplementary files 1 and 2 respectively. Reads mapping percentages for joint libraries were between 12~29%, and for serum libraries 0.03~0.9%. Between 42~53% of the 1555 mouse snoRNA reference sequences were aligned for joints and 16~26% for serum.

### Identification of DE snoRNAs using SnoRNASeq

**Figure 2:**
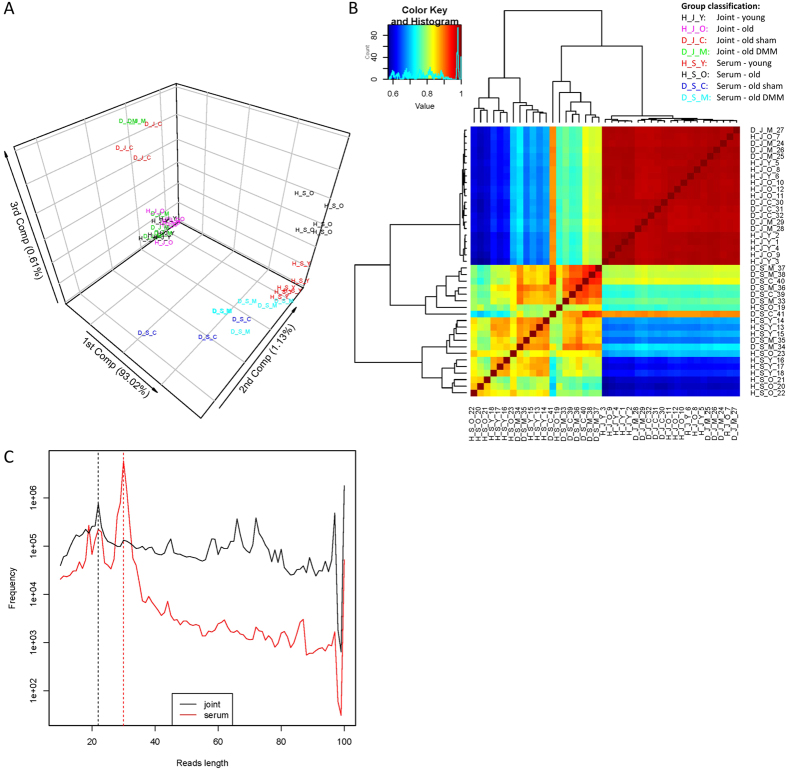
Variation data between the expressions for 41 samples. (**A**) A 3-D PCA plot of the first three components from principal component analysis of logarithm-transformed small RNA abundance data. Variance (%) associated with each principle component is depicted on the respective axis. Abbreviations; Joint: young healthy (HJY; black), old healthy (HJO; red), old sham (DJC; magenta), old DMM (DJM; light blue). Serum: young healthy (HSY; green), old healthy (HSO; dark blue), old sham (DSC; grey), old DMM (DSM; yellow). (**B**) The heat map of hierarchical clusters of correlations among samples. Pearson’s correlation coefficients were computed using logarithm transformed small RNA expression data from all known snoRNAs that were detected. (**C**) The plots of read length distribution for two representative joint and serum samples.

The 3-D PCA plot (Fig. 2A) shows that the joint samples and serum samples are clearly separated by the 1st component, which explains 93.02% of the data variation. For serum samples, the group sham and the group DMM scatter separately on the 2nd component, furthermore, based on the 3rd component a clear separation between serum samples of young-healthy and samples of old-healthy exists. For joint samples, there is also a separation between DMM and sham samples, though the separation is not as clear as shown for serum samples. In addition, 5 DMM joint samples scatter far away from other joint samples on the 3rd component. Therefore, it can be expected that disease response small RNA can be detected from this data set. The heat map of hierarchical clusters of correlations among samples (Fig. 2B) depicts that the joint and serum groups of samples are very different in snoRNA expression. In addition, joint samples correlated to each other much more closely than serum samples confirming the phenomena revealed by the PCA analysis shown in Fig. 2A. This indicates that major disease responses in the data were contributed from serum samples. A read length distribution graph was generated to highlight the constitutional difference of non-coding RNAs in joint and serum samples at 100 bp or below (Fig. 2C). For this one serum and one joint sample with approximately the same library size were chosen. The plots of the read length distribution for joint and serum samples reveal that a peak value of frequency for read length in joint samples is 22 bp (vertical, dotted black line). Therefore the corresponding reads are generally well annotated miRNAs. Another peak is noted at around 100 bp, which is consistent with non-coding RNAs whose length is a hundred bp and over. In contrast, a peak is presented at 30 bp long for serum samples (vertical, dotted red line). More than 50% of the reads are 30 bp long in the serum library (data not shown). In contrast, the joint library has just 1.14% of reads that are 30 bp long (data not shown). Such reads may come from piwi-interacting RNAs (piRNAs). There were 498–646 snoRNAs expressed in serum samples and 1068–1286 in joint samples. The DE snoRNAs between contrasts are in Table 2. These included 6 snoRNAs in young versus old joints, 5 snoRNAs in old sham versus old DMM joints. In serum we identified DE of 27 snoRNAs in young versus old serum and 18 snoRNAs in old sham versus old DMM serum.

### Validation of SnoRNASeq data using qRT-PCR

**Figure 3:**
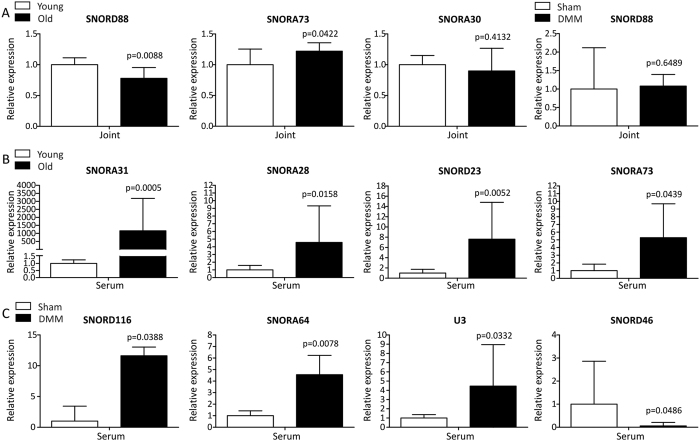
SnoRNA expression from SnoRNASeq was validated with qRT-PCR. (**A**) Gene expression patterns of SNORD88, SNORA73 and SNORA30 were confirmed in young and old joint. SNORD88 expression was verified in sham and DMM joint. (**B**) SNORA31, SNORA28, SNORD23 and SNORA73 were confirmed to be increased in old serum. (**C**) Gene expression patterns of SNORD116, SNORA64, U3 and SNORD46 were validated in sham and DMM serum. Gene expression is depicted as fold induction relative to control (i.e. young or sham). Data represents the mean + 95% CI. For statistical evaluation an independent samples t-test was performed using GraphPad Prism 5 (San Diego) on log-transformed data; p-values are indicated.

Very little is known about the roles of snoRNAs as biomarker for disease or about the functional roles of snoRNAs in mammalian cell biology. We thus could not define functional criteria to select snoRNAs for validation based on prior knowledge. To stay unbiased we thus selected snoRNAs from the RNAseq data that displayed a moderate fold significant expression difference, higher fold significant expression difference, non-significant expression difference and selected box H/ACA (SNORAs) as well as box C/D (SNORDs) snoRNAs for validation. Levels of candidate snoRNAs for further qRT-qPCR analysis were determined using the original RNA from all donors used to perform the SnoRNASeq experiment. There was good concordance between SnoRNASeq and qRT-PCR platforms (Table 2 and Fig. 3). SNORD88 was significantly decreased in young versus old joint (Fig. 3A), while SNORA73 was validated to be increased in young versus old joint (Fig. 3A). In agreement with its absence in the DE group (Table 2), SNORA30 was not differentially expressed in young versus old joint (Fig. 3A) and SNORD88 was not differentially expressed in sham versus DMM joint (Fig. 3A). SNORA31, SNORA28, SNORD23 and SNORA73 were confirmed to be significantly increased in young versus old serum (Fig. 3B) and SNORD116, SNORA64 and U3 were significantly increased in sham versus DMM serum (Fig. 3C). SNORD46 was confirmed to be significantly decreased in DMM serum as compared to sham serum (Fig. 3C).

### SNORD116 in equine serum in OA

**Figure 4:**
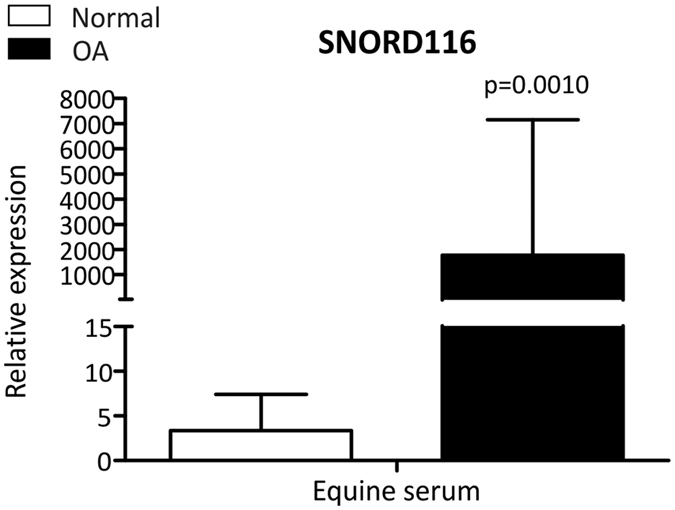
Increased gene expression of SNORD116 in equine OA serum as compared to serum from normal donors. Data represents the mean + 95% CI. For statistical evaluation an independent samples t-test was performed using GraphPad Prism 5 (San Diego) on log-transformed data; p = 0.0010.

To confirm increased SNORD116 in OA serum levels in a different species using qRT-PCR, we measured SNORD116 in equine serum samples. Normal equine donors had a Mankin’s score 1.25 ± 0.9 (mean ± 95%CI) and OA donors 7.75 ± 7.6. Synovial membrane from normal donors had a synovitis score^27^ of 3.25 ± 2.3 and OA donors 3.25 ± 3.2. There was a significant increase in serum expression of SNORD116 in OA compared to normal donors (p = 0.0010) (Fig. 4).

## Discussion

SnoRNAs are emerging as important regulators of cell functions, such as alternative splicing^28^, metabolic stress^29^ and development of disease; in cancer^6^, Prader-Willi Syndrome and autism^5^. Through expression profiling of snoRNAs using deep-sequencing we reveal novel molecular features relating to joint ageing and OA.

Whilst gene expression has been evaluated in animal models of OA, including the rat anterior cruciate transection^30^ and meniscal tear models^31^, and mouse DMM model^32^ these were primarily interrogating protein-coding genes. Furthermore, apart from the final study these experiments evaluated a single tissue, primarily articular cartilage. In musculoskeletal ageing single tissues have been investigated^13^,^14^. OA is a process that involves the whole joint as an organ; therefore we undertook our analysis on whole mouse joints, which included cartilage, meniscus, subchondral bone, and joint capsule with synovium.

Mice are considered skeletally mature at around 3-months-old (approximate equivalent of a teenaged human) while a 12-month-old mouse would signify a 40 to 50 year human^33^. Thus, to investigate the effects of joint age we used 8-month-old equivalent to 25 to 28 year human (referred to as young) and 24-month-old mice (referred to as old). To study the development of post-traumatic OA we measured OA severity histologically and analysed snoRNAs expression in joints and serum from 24-month-old mice following DMM. Limitations of this study were that we did not additionally undertake the DMM model in young mice. Additionally the ‘old’ age in young versus old contrast was not the same age as ‘old’ age in sham versus DMM. OARSI scores that were observed in the sham group (DMM surgery cohort; 24 months old) were lower than the old group (young versus old cohort; 18 months old). At this moment we cannot explain this difference, but different housing and environments of the two cohorts may have influenced this. The DMM model is a post-injury model in which the histological lesions within the affected joint are similar to those observed in human OA^17^. Mild OA-like pathology was present in the old and sham mice implying that mice at this age are in the early stages of acquiring naturally occurring OA comparable to studies of human knees at the equivalent age of approximately 40 years-old^34^. However the effect of the DMM model exacerbated these changes.

Detection of snoRNAs in serum has previously been demonstrated^12^,^35^,^36^. SnoRNAs are serum stable, and although normally resident in the nucleolus it is thought in serum they are present as unidentified protein complexes^12^. It is however not clear whether disease-associated RNAs detected in the circulation result from local tissue disturbances and cell death, or whether they are actively locally secreted via exosomes or microvesicles or are a systemic response upon local tissue damage^8^,^37^,^38^. This may even depend on the specific pathology or specific RNA species. Determining the average read-length distribution of representative joint and serum samples (Fig. 2C) revealed an unexpected finding. When looking at the read-length distribution the serum reads specifically contain a large peak of RNAs with a length of approximately 30 nt. The combined realisation that quite a large portion of the reads could not be mapped back to the used genome database, that a large portion of the piRNA class of non-coding RNAs lacks annotation in the used genome database and that piRNAs are typically 30 nt long, makes it is reasonable to think that this explains the presence of the large 30 nt peak specifically in serum. Only recently the presence of piRNAs in human blood has been described^39^ and this significant difference in the constitution of small RNAs for joint and serum samples is a phenomenon calling for further investigation.

In the young versus old serum (27 snoRNAs) more snoRNAs were significantly up-regulated compared to the old sham versus old DMM serum (18 snoRNAs) indicating ageing *per se* had a greater effect on differential snoRNA presence in serum than OA. This could be due to different ‘old’ ages in the comparisons, but also maybe expected as in ageing serum snoRNAs will be from many tissues whereas in the DMM model we are most likely highlighting primarily OA joint-related snoRNAs. As we were studying the whole joint this may be due to varying expression of snoRNAs in joint tissues, as it is known that there is tissue-specific snoRNAs expression^40^. We are unable to determine the amount each tissue type contributed to overall expression of snoRNAs. Each tissue will vary in its cellularity and hence RNA and snoRNAs content. Thus whilst a novel aspect of this study was that snoRNAs were extracted from the multiple tissues that form the joint, our approach may be less sensitive in detecting snoRNAs that change in a single tissue. However it has the advantage of determining snoRNAs that could be more globally implicated in OA. Despite the potential limitations, we identified a number of potentially interesting snoRNAs for future studies.

SNORA73 was increased in old joint and serum (Table 2, Fig. 3) and represents a potential joint ‘biological ageing’ marker. A reliable measurement of the state of ageing and a prediction of the risk of the onset of morbidity for chronic age-related diseases such as OA would be beneficial. Such a strategy could serve as a measure of ‘biological’ age and predict an age-related biological response more accurately than chronological age. SNORA64 was increased and SNORD46 was decreased in DMM serum, but both were not DE in young versus old serum, indicating these snoRNAs as possible OA markers. SNORD18 was increased in serum both in ageing and following DMM (Table 2) signifying that they are affected in ageing and also OA. It is difficult to speculate how much this snoRNA changes in age-related OA versus age from this study. Histology identified mild increase in OARSI score with age and the level of OA changes in the DMM model was mild. It would be beneficial to investigate this snoRNAs further in tissues and serum in more severe OA.

An interesting finding was an increase in SNORD38 in ageing joint. Zhang *et al*.^12^ demonstrated a strong association between serum levels of SNORD38 and severe cartilage damage, in anterior cruciate ligament (ACL) injury, enabling distinction between ACL injury patients from normal donors. They were unable to determine age effect of SNORD38 in serum from normal donors as it was undetectable. One year post-surgery there was no relationship between donor age and serum SNORD38. Our study demonstrated an increase in SNORD38 in mouse joints (but not serum) with age. The human study did not assess tissue snoRNAs and the serum samples were primarily from middle-aged donors. Our old mice group represents an equivalent older age than that in the human study, which could contribute to this disparity.

The most DE snoRNA in DMM serum was SNORD116. Additionally we demonstrated an increase in serum SNORD116 in horses with MCP OA. We have previously identified SNORD116 as increased in OA compared to normal human cartilage in an array study^15^. A loss of SNORD116 is a significant contribution to the aetiology of the neurodegenerative genetic condition Prader-Willi syndrome (PWS)^41^. This paternally imprinted disorder results in developmental delay and genetic obesity due to hyperphagia^42^. Clinical signs include short stature and low bone mineral density^43^. In a recent mouse transgenic study the loss of PWS critical region (including SNORD116) resulted in reduced bone mineral density (BMD), delayed skeletal development and reduced bone size and osteoblastic suppression^44^. Humans with OA have an increased BMD in affected joints^45^. Thus our findings not only elucidate a potential marker of OA but a snoRNA with a potential role in the pathogenesis of OA.

Previously we have identified DE snoRNAs in ageing cartilage^13^ and tendon^14^ and together with results in this study we propose that in musculoskeletal tissues snoRNAs potentially modulate the ageing process as previously described^46^. While determining the transcriptomic signature of ageing equine cartilage^13^ we found the differential expression of a number of snoRNAs associated with ageing. When comparing the differentially expressed snoRNAs between the equine and this mouse study it is important to realize that in our previous equine study we did not analyse serum or the whole joint, but specifically the articular cartilage. Overlapping differential snoRNA expression between studies was identified for SNORD113, SNORA53, SNORA48 and SNORA5. SNORA53 was decreased in old equine cartilage, but increased in old mouse serum. SNORA48 was decreased in old equine cartilage, and decreased in old DMM mouse serum. SNORA5 was increased in old equine cartilage and increased in old mouse serum. Possibly the best consistency was found for SNORD113, which was decreased in old mouse joint (and old DMM mouse joint) and equine cartilage. The apparent cross-species conservations of differentially expressed snoRNAs in ageing and OA strengthen our belief that snoRNAs could indeed be used as biomarkers. Further analysis of snoRNA expression profiles and detailed genetic studies will give new insights into novel molecular networks in musculoskeletal ageing and common mechanisms in ageing and age-related diseases such as OA.

In mammalians the majority of snoRNAs are encoded within the introns of protein coding or non-coding genes; host-genes^47^. There is evidence that genes which host snoRNAs might contribute to the aetiology of cancer through regulation of cell homeostasis and cancer biology (reviewed^6^). Potentially both the host-gene and the snoRNAs encoded within them may be important in different situations. For example growth-arrest-specific-5 (GAS-5) (hosts ten C/D box snoRNAs^48^), a non-coding RNA which accumulates in growth arrested cells, regulates cell death and proliferation by acting as a decoy hormone response element for glucocorticoid receptors thereby inhibiting gene upregulation by activated glucocorticoid receptors^49^. Interesting snoRNA host-genes were identified in this study including transforming growth factor β regulated gene 4, sorting nexin 5 and collagen type 1 (with roles in joint homeostasis). Therefore, an alteration of snoRNA expression may result from changes in transcriptional activity of the host-gene related to joint homeostasis or disease.

## Conclusion

Our results implicate specific changes in snoRNA abundance in joint ageing (SNORD88 and SNORD38 were respectively decreased and increased) and OA suggesting the potential use of snoRNAs such as SNORA73 and SNORD23 as a novel biomarker for joint ageing, SNORA64, SNORD46 and SNORD116 for OA, SNORD18 for ageing and OA.

## Additional Information

**How to cite this article:** Steinbusch, M. M. F. *et al*. Serum snoRNAs as biomarkers for joint ageing and post traumatic osteoarthritis. *Sci. Rep.* **7**, 43558; doi: 10.1038/srep43558 (2017).

**Publisher's note:** Springer Nature remains neutral with regard to jurisdictional claims in published maps and institutional affiliations.

## Supplementary Material

Supplementary Dataset 1

Supplementary Dataset 2

## Figures and Tables

**Table 1:** Oligonucleotides sequences used in qRT-PCR.

Name	Sequence (5′-3′)	*T*m (°C)
RTQ primer poly(A) cDNA synthesis	CGAATTCTAGAGCTCGAGGCAGGCGACATGGCTGGCTAGTTAAGCTTGGTACCGAGCTCGGATCCACTAGTCCTTTTTTTTTTTTTTTTTTTTTTTTTVN	75
Snora28	CATGAGACAAGCCGTTATATAGGC	50
Snora30	TGTACCAGTGGCAGCTGTTACTC	50
Snora31	CTTTGTGGCAGTTCAGATTGAATTAG	50
Snora64	GTGGCCTCTCTTGCCTAGAG	65
Snora73	ACAGTGACTGAGGAGGCAAAC	50
Snord46	AATGCAAGGACTTGTCATAGTTACAC	50
Snord85	TTAGACCAGAGGTCGATGATGAG	50
Snord88	ACCTTTGACACCTGGAGATCTGA	50
Snord116	TGTACCGCCACTCTCATCGG	65
U2	TGGTATTGCAGTACCTCCAGGAACG	55
U3	AGTGAGAGGGAGAGAACGCGGTC	55
U6	GATGACACGCAAATTCGTGAAGCGTTC	55
RTQ-UNIr-50	AATTCTAGAGCTCGAGGCAGG	50
RTQ-UNIr-55	CGAATTCTAGAGCTCGAGGCAGG	55
RTQ-UNIr-65	CTAGAGCTCGAGGCAGGCGACATGGCTGGC	65

**Table 2:** Differentially expressed snoRNAs between contrasts including host gene identification.

Contrast	Gene ID	Name	Log_2_ FC	FDR	Host Gene
Young vs. Old joint	ENSMUSG00000098372	SNORD113	−0.92	0.05	Predicted gene
	ENSMUSG00000065016	SNORA3	−0.80	0.01	Ribosomal protein L27A
	ENSMUSG00000080352	SNORD88	−0.55	0.00	RIKEN cDNA 2410002F23 gene
	ENSMUSG00000064380	SNORA73	0.56	0.03	Ribosomal protein SA
	ENSMUSG00000087883	SNORA17	1.21	0.05	Contactin associated protein-like 5B
	ENSMUSG00000064536	SNORD38	1.49	0.05	PDZ domain containing 4
Young vs. Old serum	ENSMUSG00000089255	SNORA78	−2.29	0.01	Ribosomal protein S2
	ENSMUSG00000080396	SNORD111	−1.38	0.01	Splicing factor 3b, subunit 3
	ENSMUSG00000065883	U3	−2.53	0.00	Predicted gene
	ENSMUSG00000064500	SNORD95	1.16	0.01	Guanine nucleotide binding protein, beta polypeptide 2 like 1
	ENSMUSG00000095118	SNORD14D	1.23	0.00	Heat shock protein 8
	ENSMUSG00000065219	SNORD32A	1.55	0.04	Ribosomal protein L13A
	ENSMUSG00000088678	SNORA17	1.90	0.03	Predicted gene
	ENSMUSG00000064938	miR3068/SNORA58	1.96	0.00	AlkB, alkylation repair homolog 1
	ENSMUSG00000065282	SNORA18	1.99	0.00	Predicted gene
	ENSMUSG00000064387	SNORA73A	2.02	0.01	Regulator of chromosome condensation 1
	ENSMUSG00000064602	SNORA41	2.12	0.00	Eukaryotic translation elongation factor 1 beta 2
	ENSMUSG00000088670	SNORA31	2.14	0.03	Regulatory associated protein of MTOR, complex 1
	ENSMUSG00000064918	SNORD18	2.18	0.02	Ribosomal protein L4
	ENSMUSG00000065158	SNORA73	2.26	0.02	ERO1-like beta
	ENSMUSG00000064493	SNORA28	2.39	0.03	Eukaryotic translation initiation factor 5
	ENSMUSG00000077167	SNORA53	2.43	0.00	Solute carrier family 25
	ENSMUSG00000064495	SNORA5	2.49	0.00	Transforming growth factor beta regulated gene 4
	ENSMUSG00000064569	SNORD71	2.56	0.00	Adaptor-related protein complex 1, gamma 1 subunit
	ENSMUSG00000064791	SNORD14E	2.63	0.00	Heat shock protein 8
	ENSMUSG00000077714	SNORD17	2.76	0.00	Sorting nexin 5
	ENSMUSG00000065353	SNORD73B	2.79	0.00	Regulator of chromosome condensation 1 and an lnc
	ENSMUSG00000064696	SNORD20	3.09	0.00	Nucleolin
	ENSMUSG00000065778	SNORA55	3.10	0.02	Poly(A) binding protein, cytoplasmic 4
	ENSMUSG00000080478	SNORD23	4.42	0.00	Glioma tumor suppressor candidate region gene 2
	ENSMUSG00000095176	SNORA44	5.65	0.00	Hypothetical protein PNAS-123
	ENSMUSG00000088040	SNORA17	12.14	0.00	Hypothetical protein MGC16037, homology human collagen alpha 2
	ENSMUSG00000065852	SNORA2	13.40	0.00	Hypothetical protein, 152aa
Sham vs. DMM joint	ENSMUSG00000064655	SNORA55	−0.88	0.04	Poly(A) binding protein, cytoplasmic 4
	ENSMUSG00000098971	SNORD113	−0.99	0.04	Lnc-NA imprinted and accumulated in nucleus
	ENSMUSG00000064858	SNORA43	−1.12	0.04	Lnc- small nucleolar RNA host gene 7
	ENSMUSG00000065105	SNORA29	−1.22	0.04	T-complex protein 1
	ENSMUSG00000077191	SNORA64	−1.62	0.04	Small nuclear ribonucleoprotein N
Sham vs. DMM serum	ENSMUSG00000096017	SNORD116	10.56	0.01	Predicted gene
	ENSMUSG00000064938	miR3068	3.31	0.03	AlkB, alkylation repair homolog 1
	ENSMUSG00000089014	SNORA36	3.05	0.01	Dyskerin
	ENSMUSG00000077222	SNORA66	2.78	0.05	Ribosomal protein L5
	ENSMUSG00000064918	SNORD18	2.21	0.01	Ribosomal protein L4
	ENSMUSG00000064400	U3	1.92	0.01	Predicted gene
	ENSMUSG00000089417	U90	1.90	0.01	Importin
	ENSMUSG00000077709	SNORA64	1.85	0.04	Small nuclear ribonucleoprotein N
	ENSMUSG00000065734	SNORD49A	−1.03	0.05	RIKEN cDNA 2410006H16 gene
	ENSMUSG00000064450	SNORD68	−1.06	0.04	Ribosomal protein L13
	ENSMUSG00000064844	SNORD58	−1.18	0.01	Ribosomal protein L17
	ENSMUSG00000064453	SNORD21	−1.22	0.01	Ribosomal protein L5
	ENSMUSG00000065281	SNORD27	−1.31	0.01	Small nucleolar RNA host gene 1
	ENSMUSG00000064871	SNORD58B	−1.40	0.05	Ribosomal protein L17
	ENSMUSG00000087819	SNORA48	−1.41	0.04	Nucleosome assembly protein 1-like 1
	ENSMUSG00000064751	SNORD46	−1.46	0.01	Ribosomal protein S8
	ENSMUSG00000077756	SNORD90	−1.48	0.01	Ring finger and CCCH-type zinc finger domains 2
	ENSMUSG00000089093	SNORD11	−1.86	0.03	NOP58 ribonucleoprotein

Explanation: positive Log_2_ FC = increased in old or DMM. Negative Log_2_ FC = is decreased in old or DMM.
